# Hodgkin Lymphoma has a seasonal pattern of incidence and mortality that depends on latitude

**DOI:** 10.1038/s41598-017-14805-y

**Published:** 2017-11-02

**Authors:** Sven Borchmann, Horst Müller, Andreas Engert

**Affiliations:** German Hodgkin Study Group (GHSG), Department I of Internal Medicine, University Hospital Cologne, Cologne, Germany

## Abstract

Seasonal variations in incidence and mortality after a Hodgkin lymphoma (HL) diagnosis have been previously described with partly conflicting results. The goal of this analysis is to provide a comprehensive analysis of these seasonal variations. In total, 41,405 HL cases diagnosed between 1973 and 2012 in the 18 Surveillance, Epidemiology, and End Results registries were included. Cosinor analysis and Cox proportional-hazards models were employed to analyze seasonality of incidence and mortality, respectively. HL shows a sinusoid seasonal incidence pattern (p < 0.001). Estimated incidence in March is 15.4% [95%-CI: 10.8-20.0] higher than in September. This sinusoid pattern is more pronounced at higher latitudes (p = 0.023). The risk of dying within the first three years after a HL diagnosis in winter is significantly increased compared to a HL diagnosis in summer at higher latitudes (HR = 1.082 [95%-CI: 1.009-1.161], p = 0.027). Furthermore, increasing northern latitude increases the additional mortality risk conferred by a diagnosis in winter (p_interaction_0.033). The seasonality patterns presented here provide epidemiological evidence that Vitamin D might play a protective role in HL. Further evidence on the direct association between Vitamin D levels and the clinical course of HL needs to be collected to advance the understanding of the role of Vitamin D in HL.

## Introduction

Hodgkin lymphoma (HL) is among the hematological malignancies with the best prognosis. Ongoing efforts to improve treatment efficacy have resulted in 5-year overall survival (OS) rates of above 95% being reported in clinical trials in various disease stages^1–4^. Despite this, pathogenesis of HL is poorly understood and there are potential unknown risk factors that might affect HL incidence and mortality. Examining seasonal patterns of incidence and mortality might be helpful in improving the understanding of both, the pathogenesis of the disease and the influence of seasonally variable risk factors on incidence and mortality.

Seasonal variations in incidence and mortality of HL have been previously described in small datasets^5–10^ with partly conflicting patterns and results. Interestingly, all published studies examining these patterns were performed in fairly northern countries such as Norway, Sweden and northern parts of England or Scotland.

The goal of the present study is to use Surveillance, Epidemiology, and End Results (SEER) registry data to perform a comprehensive analysis of seasonality patterns in incidence and mortality of HL. By using a large and geographically diverse dataset, it is possible to analyze geographic variations of seasonality patterns, which might allow better insights into possible causes and mechanisms of this malignancy.

## Methods

All data was obtained from the SEER incidence database using the November 2014 submission^11^. The SEER program provides population based cancer registry data from 18 SEER registries covering approximately 28% of the U.S. population. A detailed description of the SEER registries, methods of data collection and follow-up is available through the SEER website^12^.

To calculate the number of incident cases in each month, the SEER*Stat software^13^ was used to select all HL cases diagnosed between 1973 and 2012 in the 18 SEER registries. This selection resulted in 50,179 actively followed cases. All death certificate and autopsy only cases (n = 281) were excluded. Following that, all cases without known month of diagnosis (n = 257) were excluded. The SEER registries “Alaska natives” (n = 17) and “Rural Georgia” (n = 57) were excluded due to low case numbers. Finally, some cases had no information available for age of diagnosis (n = 5) and Ann-Arbor stage (n = 8,157) and were excluded. This selection process resulted in 41,405 cases. A CONSORT flowchart visualizing the selection process is provided as Supplementary Figure 1. The characteristics of the cases included in the analysis are described in Table 1 and are representative of the general HL population.

**Table 1:** Patient characteristics and number of cases for each subgroup in the dataset.

		No. of cases
Total		41405 (100%)
Sex	Male	22708 (54.8%)
	Female	18697 (45.2%)
Histological subtype	Lymphocyte rich	1371 (3.3%)
	Mixed cellularity	6172 (14.9%)
	Lymphocyte depleted	684 (1.7%)
	Nodular sclerosis	24631 (59.5%)
	NLPHL	1619 (3.9%)
	Not otherwise specified	6928 (16.7%)
Age group	0–19	5352 (12.9%)
	20–29	9869 (23.8%)
	30–39	7865 (19.0%)
	40–49	5610 (13.6%)
	50–59	4202 (10.1%)
	60–69	3647 (8.8%)
	≥70	4860 (11.8%)
Ann-Arbor stage	I	8824 (21.3%)
	II	16309 (39.4%)
	III	8537 (20.6%)
	IV	7735 (18.7%)
Year of diagnosis	<1990	4608 (11.1%)
	≥1990 & <2000	9024 (21.8%)
	≥2000 & <2010	21278 (51.4%)
	≥2010	6495 (15.7%)
Latitude	<34.19°N	7932 (19.2%)
	≥34.19°N & <38.05°N	12737 (30.8%)
	≥38.05°N & <41.68°N	10152 (24.5%)
	≥41.68°N	10584 (25.5%)

NLPHL denotes nodular lymphocyte-predominant Hodgkin lymphoma.

All calculations were performed using the software R^14^ and R Commander^15,16^. Survival analysis and cosinor analysis were performed using the R-packages survival^17^ and cosinor^18^, respectively. All p-values were calculated as two-sided p-values. P-values < 0.05 were considered to be statistically significant in all statistical tests employed. All p-values < 0.001 are presented as such. All confidence intervals presented are 95%-intervals. With regard to the subgroup analysis of the seasonal incidence pattern, correction for multiple testing was performed according to the Bonferroni-Holm method^19^ and adjusted p-values are indicated with p_adj_ where applicable.

### Seasonality of incidence

The date of diagnosis was defined as the month, day and year the HL was first diagnosed clinically or microscopically by a recognized medical practitioner^12^. Cases were grouped according to month of diagnosis. The number of cases per month were adjusted for differences in the length of the respective month by dividing each month’s case count by the number of days in that month, with February having 28.25 days, and multiplied by the average number of days across all months (30.4375 days). Subsequently, adjusted case counts were standardized, so that the average month had an incidence of 1. This was done by dividing each month’s adjusted case count by the average case count across all months. Cosinor analysis was performed to examine seasonality in the incidence data using the standardized monthly incidence as input. A detailed description of cosinor analysis is provided by Nelson *et al*. and Tong *et al*.^20,21^. In order to examine seasonality in various subgroups, the cases were stratified by sex, histological subtype, age group, Ann-Arbor stage, year of diagnosis and latitude quartiles. Age at diagnosis was sorted into groups aged 0–19, 20–29, 30–39, 40–49, 50–59, 60–69 and >70, respectively. For each case, the location of diagnosis was available from the SEER database at county level. Latitude quartiles were defined by sorting all counties by their respective latitude and allocating the counties into quartiles by latitude. Data on latitude for the single counties was provided by the U.S. Census Bureau^22^. The peak incidence month is defined as the month closest to the incidence peak estimated by the cosinor model. Amplitude is defined as the amplitude estimated by the cosinor model.

Cases were subdivided into cases diagnosed in the northern half (latitude ≥38.05°N) and southern half (latitude <38.05°N) of counties. Subsequently, a dichotomous variable representing the location of diagnosis (northern half vs. southern half of counties) was included into the cosinor model and its influence on the amplitude tested.

### Seasonality of Mortality

In order to analyze seasonal differences in mortality, a Cox proportional-hazards model was employed^23^. Seasonality was first tested by grouping the month of diagnosis into a dichotomous variable and, thus, dividing the cases into those diagnosed during the winter (September to February) and summer half year (March to August). This dichotomization was chosen on the basis of the meteorological definition of the seasons^24^. In the present study, the summer half year comprises the meteorological spring and summer and the winter half year comprises the meteorological autumn and winter. All known risk factors for a poor outcome of HL available from the SEER database were included into the model to control for their effect: Age at diagnosis, histological subtype, year of diagnosis, sex and Ann-Arbor stage.

Follow-up was censored at three years to focus on short term mortality, taking into consideration the hypothesis that mortality differences by season of diagnosis are less pronounced with increasing follow-up, as patients live through more seasons in total, thus decreasing the relative exposure to a specific season that might be a risk factor. A model with 5 years of follow up was fitted as well in order to increase robustness of results. Analysis was firstly performed for all cases together and then stratified by the above mentioned subdivision into the northern vs. southern half of counties.

In order to evaluate a possible effect of latitude on the difference in mortality after a diagnosis in winter versus summer, an additional Cox proportional-hazards model was fitted. This model included a multiplicative interaction term between the dichotomous variable indicating the season of diagnosis and the continuously modelled latitude of the county the case was diagnosed in.

In order to test for robustness of results with respect to dividing the year into two half years at specific months, a further Cox proportional-hazards model was fitted including a sinusoid term x_1_ that was calculated with the following formula:

1 $${x}_{1}=\,cos[2\pi (\frac{M-{M}_{max}}{12})]$$

where M is the month of diagnosis (e.g. January = 1, February = 2…) and M_max_ is the month with the maximum risk. For example, for M_max_ = 3, this term assigns March the highest possible seasonal risk 1 and September the lowest −1. For M_max_ = 4, the seasonal risk term shifts forward by one month so that April is assigned the highest seasonal risk 1 and October the lowest −1 and so on. Substituting this seasonal risk term for the dichotomized season of diagnosis variable in the Cox proportional-hazards model described above and iteratively d**e**termining M_max_ so that β_1_ in the Cox proportional-hazards model is maximized makes it possible to find the highest risk month that describes the true peak and trough of seasonal mortality risk in the data best. A detailed explanation of the concept is given by Efird *et al*.^25^.

### Data availability

The data analysed in the current study is available from the SEER registries at https://seer.cancer.gov/registries/.

## Results

### Seasonal incidence pattern

**Figure 1:**
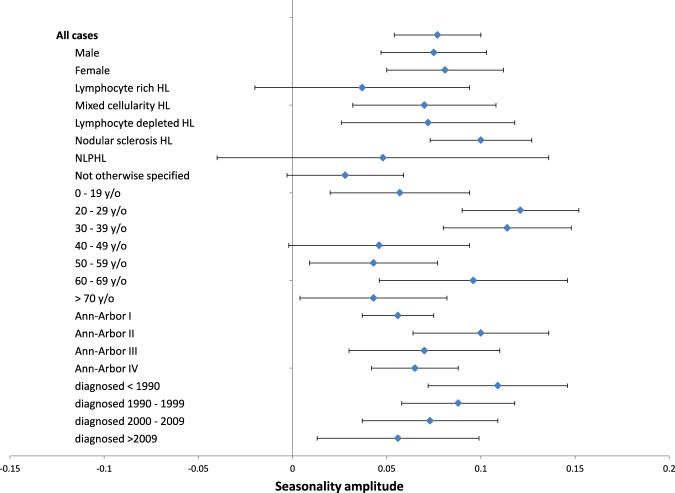
Forest plot of the subgroup analysis of seasonality of incidence The seasonality amplitude is shown on the x-axis. Subgroups are shown on the y-axis. The estimated seasonality amplitude with 95% confidence intervals is shown for each subgroup.

Looking at all incident cases together, a highly significant (p < 0.001) seasonal incidence pattern emerged with a peak around March and a trough around September. The estimated amplitude of seasonality was 0.077, indicating that the incidence around September is roughly 15% lower than around March. Table 2 shows the raw, adjusted and normalized incidence for each month for the whole dataset. Figure 1 shows a forest plot with all subgroup analyses. The seasonal pattern was equally present in male and female cases. Stratified by histological subtype, only cases of the mixed cellularity subtype (p_adj_ = 0.027), the nodular sclerosis subtype (p_adj_ = 0.027) and the lymphocyte depleted subtype (p_adj_ = 0.027) showed a significant seasonal incidence pattern when analyzed separately, while the lymphocyte rich subtype (p_adj_ = 0.412), the not otherwise specified (NOS) subtype (p_adj_ = 0.244) and the nodular lymphocyte predominant (NLPHL) subtype (p_adj_ = 0.412) did not. Despite that, there was a clear trend towards seasonality in all histological subgroups. Stratification by age group revealed a significant seasonal incidence pattern for all age groups, apart from the age groups 40–49 (p_adj_ = 0.244), 50–59 (p_adj_ = 0.084) and >70 (p_adj_ = 0.160). The amplitude was particularly high for the age groups 20–29 (0.121), 30–39 (0.114) and 60–69 (0.096), coinciding with higher HL incidence age groups^26^. Furthermore, stratification by Ann-Arbor stage and year of diagnosis revealed significant seasonality across these subgroups. Despite some non-significant results after adjusting for multiple testing, there was a clear trend towards seasonality in all subgroups (Fig. 1). The non-significant seasonality in some subgroups might be due to lower case numbers in the respective groups.

**Table 2:** Raw, adjusted and normalized incidence of Hodgkin lymphoma for each month in the dataset.

Month	Case count	Adjusted case count	Normalized incidence
January	3698	3631	1.052
February	3454	3721	1.078
March	3846	3776	1.094
April	3701	3755	1.088
May	3412	3350	0.970
June	3466	3517	1.019
July	3283	3223	0.934
August	3318	3258	0.944
September	3154	3200	0.927
October	3355	3294	0.954
November	3298	3346	0.969
December	3420	3358	0.973

The adjusted case count is rounded to the nearest integer.

**Figure 2:**
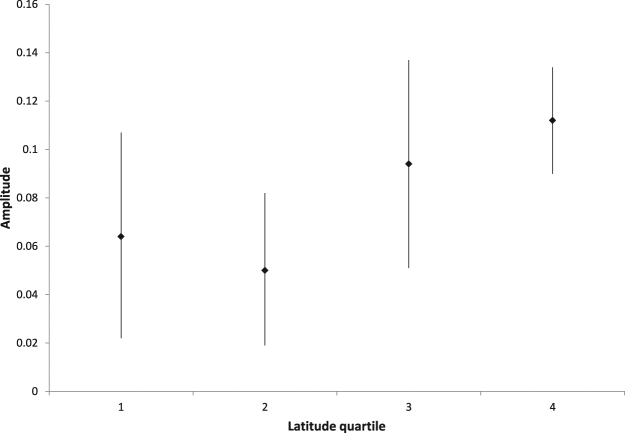
Relationship between latitude quartile and estimated seasonality amplitude Latitude quartiles are shown on the x-axis. The first, second, third and fourth latitude quartiles include all counties south of 34.19°N, between 34.19°N and 38.05°N, between 38.05°N and 41.68°N and north of 41.68°N, respectively. The estimated seasonality amplitude of the respective quartile is shown on the y-axis with 95% confidence intervals of the estimated amplitude.

Looking at the stratification by latitude quartiles, all quartiles showed significant seasonality patterns when analyzed separately (Fig. 2). The amplitude was dependent on the latitude. Cases diagnosed at a latitude <38.05°N (southern half of counties) had an amplitude of 0.055 ([0,026; 0.084], p < 0.001) compared to 0.102 ([0.073; 0.131], p < 0.001) for those diagnosed at a latitude ≥38.05°N (northern half of counties). The difference of 0.05 ([0.01; 0.09], p = 0.023) between these groups was statistically significant.

### Impact of seasonality and latitude on outcome

The results of the Cox proportional-hazards model for overall survival after a HL diagnosis with season of diagnosis as a dichotomous variable are given in Table 3. Looking at all cases together, being diagnosed in winter does not significantly increase the risk of dying compared to a diagnosis in summer within 3 years after diagnosis (HR = 1.030 [0.981, 1.081], p = 0.234). However, when analyzed stratified by latitude, cases from the northern half (latitude ≥38.05°N) of counties exhibited an increased risk of death when the diagnosis of HL was made in winter (HR = 1.082 [1.009, 1.161], p = 0.027). In contrast, cases from the southern half of counties (latitude <38.05°N) showed no increased risk (HR = 0.990 [0.926, 1.059], p = 0.772). Confirmatory analysis using 5-year survival data showed similar results (Table 3).

**Table 3:** Results of the estimated Cox proportional-hazards model for all cases and stratified by latitude.

		Hazard Ratio	Lower 95% CI	Upper 95% CI	p
3-year survival	All cases	1.030	0.981	1.081	0.234
	**≥38.05°N**	**1.082**	**1.009**	**1.161**	**0.027**
	<38.05°N	0.990	0.926	1.059	0.772
5-year survival	All cases	1.027	0.983	1.073	0.238
	**≥38.05°N**	**1.073**	**1.007**	**1.144**	**0.029**
	<38.05°N	0.991	0.932	1.053	0.764

Age at diagnosis, histological subtype, year of diagnosis, sex and Ann-Arbor stage were included into the model as known risk factors to control for their effect. Hazard ratio is the hazard ratio for overall mortality in the first three or five years after the HL diagnosis when being diagnosed in winter (September to February) versus summer (March to August). 95% confidence intervals of the hazard ratio are given. Significant results are shown in bold.

To further examine the relationship between latitude and the increased risk of death after a winter or summer diagnosis, a multiplicative interaction term between the dichotomous season variable and the latitude each case was diagnosed at (in 10° steps) was included into the Cox proportional-hazards model described above. The hazard ratio for this interaction term was 1.119 ([1.009; 1.241], p = 0.033) using 3 year survival data and, thus, significantly greater than 1. This interaction term indicates that increasing northern latitude increases the additional mortality risk after a winter versus summer diagnosis. Confirmatory analysis using 5-year survival data showed similar results with a hazard ratio for the interaction term of 1.105 ([1.005; 1.214], p = 0.039).

In a final Cox proportional-hazards model with 3-year survival data, robustness of the cutoff months for dividing the year into a summer and a winter half year was assessed. A seasonal risk term, calculated as stated in the methods section, substituted the dichotomized season of diagnosis variable. Table 4 shows the estimated hazard ratios for the seasonal risk term for various M_max_, each reflecting a different peak risk month for cases diagnosed in the northern half (latitude ≥38.05°N) of counties. The hazard ratio was highest for the seasonal risk term with M_max_ = 11 indicating the seasonal risk term implying November as the peak mortality risk month describes the seasonal mortality risk best. For M_max_ = 11, the seasonal risk term takes positive values for the months September to January, a neutral value of 0 for the months February and August and negative values for the months March to July. These intervals coincide with the chosen dichotomization of the year into a summer and a winter half year employed in the previous models. Note how β_1_ and, thus, the hazard ratio is lowest for M_min_ = 5, reflecting the sinusoid property of the seasonal risk term. For M_min_ = 5, the seasonal risk term is 1 for cases diagnosed in May, thus implying the lowest seasonal mortality risk in May. Finally, we tested a multiplicative interaction term as described above in this model, this time indicating the interaction between latitude (in 10° steps) and the seasonal risk term with M_max_ = 11. The hazard ratio for this interaction term was 1.103 ([1.025; 1.187], p = 0.009) and, thus, significantly greater than 1.

**Table 4:** Hazard ratio for the seasonal risk term for various peak risk months.

Peak risk month	Hazard Ratio	Lower 95% CI	Upper 95% CI	p
January	1.023	0.973	1.075	0.380
February	0.992	0.944	1.042	0.748
March	0.965	0.919	1.014	0.155
April	0.947	0.902	0.995	0.032
May	0.943	0.897	0.991	0.021
June	0.954	0.908	1.003	0.065
July	0.978	0.931	1.028	0.380
August	1.008	0.956	1.060	0.748
September	1.036	0.987	1.089	0.155
October	1.056	1.005	1.109	0.032
November	1.060	1.009	1.114	0.021
December	1.048	0.997	1.102	0.065

The Cox proportional-hazards model included only cases diagnosed north of 38.05°N. The hazard ratios for different peak risk months are shown. A detailed explanation of the calculation of the seasonal risk term is given in the methods section. 95% confidence intervals of the hazard ratios are given.

## Discussion

Here, we report the most comprehensive analysis of seasonal incidence and mortality patterns in HL performed to date and present several new findings.

The overall seasonal incidence pattern of HL in the northern hemisphere has a peak around March and a trough around September. Douglas and coworkers looked at seasonal incidence patterns for the different histological HL subtypes demonstrating seasonality for the nodular sclerosis subtype and the nodular lymphocyte predominant subtype^10^. We found similar results for the nodular sclerosis subtype and, in addition to that, a significant seasonal incidence pattern for the mixed cellularity and the lymphocyte depleted subtypes. The pattern described by Douglas *et al*.^10^ for the nodular lymphocyte predominant subtype was not significant in the present study, although there was a trend towards seasonality in our data as well. Non-significance might be the result of lower case numbers in this rare subgroup. Neilly *et al*.^5^ also performed a stratified analysis of seasonal incidence patterns by histological subtype and, very similar to our analysis, also found a significant seasonal incidence pattern for the mixed cellularity and the nodular sclerosis subtypes.

A stratified analysis by age group was undertaken by Chang *et al*.^8^, however, a comparison of the amplitude of the seasonality pattern in the different age groups was not performed. In addition, age groups were defined more broadly than in our analysis, possibly due to lower case numbers. Interestingly, the seasonal incidence pattern was strongly pronounced for the age groups 20–29 and 30–39. An overall incidence peak was observed in these age groups largely associated with Epstein-Barr virus (EBV) negative cases^27^. Data on EBV association are not provided in the SEER database, but one can assume that EBV status is an important factor in determining seasonal incidence patterns and that the seasonal incidence patterns vary between EBV positive and EBV negative cases. We were also able to show that the seasonal incidence pattern is more pronounced at higher latitudes.

We found a significantly increased risk of death in the first three years after a diagnosis of HL made in winter as compared to summer at higher latitudes. Seasonal differences in mortality risk after a diagnosis of HL have previously been described by Porojnicu *et al*.^7^ although the authors only found a lower risk of mortality after being diagnosed in autumn versus winter. In our study, the increased risk of mortality after a winter diagnosis was confined to counties at higher latitudes (>38.05°N). All seasonal patterns of mortality or incidence have so far been described in fairly northern countries of high latitude in Europe^5–10^. We were able to show that the increased risk of dying after a winter diagnosis of HL is dependent on the latitude the diagnosis was made at in a way that a more northern latitude increases the risk of dying after being diagnosed with HL in winter. Robustness of our results is shown in an additional model employing a sinusoid seasonal risk term. This model shows November to be the least favorable month and May as the most favorable month of diagnosis with regard to subsequent mortality risk.

**Figure 3:**
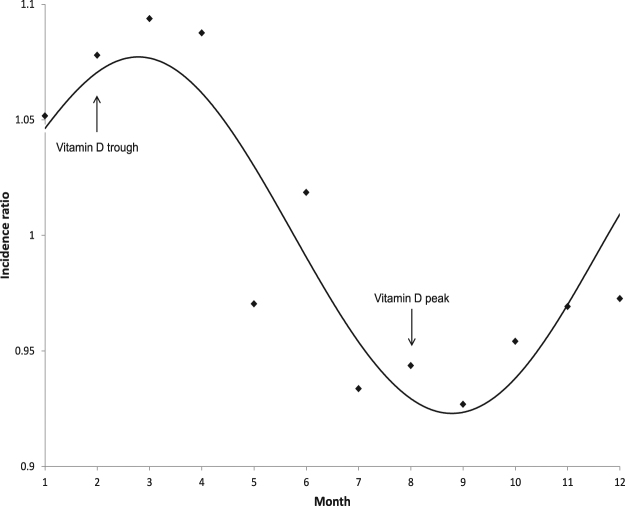
Illustration of the relationship between the peak and trough of vitamin D levels in humans in the northern hemisphere and the overall seasonality pattern of HL incidence The months of the year are shown on the x-axis. The ratio of the respective month’s incidence and the average incidence of all months are shown on the y-axis. The line indicates a fitted curve through the data as estimated by the cosinor model.

We hypothesize that seasonal differences in vitamin D levels mediated by ultraviolet radiation (UVR) are at least in part responsible for the seasonal patterns described here. The seasonal pattern of vitamin D levels in humans is also more pronounced at higher latitudes^28,29^. Strikingly, the seasonal incidence pattern observed in HL cases almost exactly resembles the seasonal pattern of vitamin D levels in humans^30^ (Fig. 3). In addition, the occurrence of a more pronounced seasonal incidence pattern among young patients could be a result of the higher seasonal variation in Vitamin D levels that has been described in this age group^31,32^. Recent epidemiological studies from Australia and the United States showed an increased incidence of HL with longer distances from the equator or lower annual ambient UVR, respectively^33,34^. Furthermore, increased ambient UVR was found to be likely protective, reducing HL incidence in a recent meta analysis^35^. Interestingly, a recent study that examined incidence of HL in Mediterranean countries found a higher HL incidence in countries bordering the northern Mediterranean Sea compared to those bordering the southern Mediterranean Sea^36^. However, these differences could also be explained by differences in sociodemographic characteristics or cancer registry methodology between these countries. A low Vitamin D status has been described as a risk factor for both, developing and suffering from a poor prognosis after a diagnosis of other hematological malignancies. Examples are follicular lymphoma^37^ or chronic lymphocytic lymphoma^38^. New treatments for HL, such as immune checkpoint inhibitors^39,40^, might be partially dependent on patients having a functioning immune system. It is known that Vitamin D supports various immune system functions and can act anti-proliferative in various hematological cancers^41,42^.

Other causes of the seasonality described here are certainly possible, for example seasonality of an infectious agent, such as EBV, causing some HL cases. This would, however, not explain the observed seasonal differences in mortality and would contradict the finding that the seasonal incidence pattern of HL is, in fact, strongest in age groups with a fairly high proportion of EBV negative cases. Another possible explanation for the seasonal incidence pattern could be an increase in diagnoses of HL in late winter, due to the coincidence of the common cold season and an increase in consultations resulting in higher health care exposure and consultations for longer lasting reasons like lymph node swellings being postponed until after the holiday season. Lower incidence numbers in late summer could be explained by the summer holiday season. However, these alternative explanations do not explain the observed mortality pattern.

A potential shortcoming of our study is that many HL relevant variables and known risk factors are not available from the SEER program. Therefore it is possible that some variables exist that might at least partly explain the observed incidence pattern or increase mortality after a diagnosis in winter. For example, bulky disease, especially in the mediastinum is a known risk factor in HL^4^ and might be more symptomatic in winter. Likewise, data to calculate the International Prognostic Score (IPS), a widely accepted score for risk stratification of HL, is not available. However, by including all available potential risk factors into the Cox proportional-hazards models used to estimate seasonal differences in mortality, we tried to control for potential confounders as good as possible with the available data. Additionally, we compared available disease and patients characteristics between the highest and lowest risk months for mortality (November vs. May) (Supplementary Table 1) and the peak and trough months for incidence (March vs. September) (Supplementary Table 2). Disease and patient characteristics were not different between the compared months.

In conclusion, we found a striking seasonal pattern of incidence and mortality in HL. Further studies are needed in order to better understand the reasons for the seasonal patterns described here.

## Electronic supplementary material

Supplementary Tables and Figure
