# Supplementary material for: Hodgkin Lymphoma has a seasonal pattern of incidence and mortality that depends on latitude
Source: Sci Rep. 2017 Nov 2;7:14903. doi: 10.1038/s41598-017-14805-y (PMC5668282; doi:10.1038/s41598-017-14805-y)
Supplement: Supplementary file 1 — Supplementary Tables and Figure [file 41598_2017_14805_MOESM1_ESM.pdf]

Hodgkin Lymphoma has a seasonal pattern of incidence and mortality that depends on latitude

Sven Borchmann[1*], Horst Müller[1], Andreas Engert[1]

[1]German Hodgkin Study Group (GHSG), Department I of Internal Medicine, University Hospital Cologne, Cologne, Germany

Supplementary Table 1

Variable		Diagnosed in September (incidence trough)	Diagnosed in March (incidence peak)	P (difference)
**Age (mean)**		40.98	40.56	0.383
**Year of diagnosis**	<1990	10.6%	10.9%	0.628
	1990-1999	21.2%	22.0%	
	2000-2009	51.3%	51.2%	
	>2010	16.9%	15.9%	
**Sex**	female	44.1%	46.1%	0.100
	male	55.9%	53.9%	
**Histological subtype**	NS	59.1%	59.5%	0.522
	MC	14.1%	14.7%	
	LR	3.0%	3.1%	
	LD	1.6%	1.5%	
	NLPHL	4.0%	4.6%	
	NOS	18.1%	16.6%	
**Ann-Arbor stage**	I	21.8%	20.3%	0.154
	II	39.0%	39.9%	
	III	19.9%	21.5%	
	IV	19.3%	18.3%	

Supplementary Table 1 shows available disease and patient characteristics for patients diagnosed in September (incidence trough) and March (incidence peak). Age was compared using a two-sided T-

test. Discrete variables (sex, year of diagnosis, histological subtype, Ann-Arbor stage) were compared using the Chi-square test. NS: Nodular sclerosis. MC: Mixed cellularity. LR: Lymphocyte-rich. LD: Lymphocyte-depleted. NLPHL: Nodular lymphocyte-predominant Hodgkin lymphoma. NOS: Not otherwise specified.

Supplementary Table 2

Variable		Diagnosed in May (mortality low)	Diagnosed in November (mortality high)	P (difference)
**Age (mean)**		40.60	40.67	0.887
**Year of diagnosis**	<1990	12.3%	11.6%	0.752
	1990-1999	21.8%	22.0%	
	2000-2009	50.0%	50.9%	
	>2010	15.9%	15.5%	
**Sex**	female	44.6%	45.2%	0.620
	male	55.4%	54.8%	
**Histological subtype**	NS	59.7%	59.7%	0.977
	MC	14.9%	14.7%	
	LR	3.1%	3.4%	
	LD	1.9%	1.8%	
	NLPHL	4.0%	4.2%	
	NOS	16.4%	16.2%	
**Ann-Arbor stage**	I	21.9%	21.5%	0.972
	II	38.5%	38.4%	
	III	20.4%	20.7%	
	IV	19.2%	19.4%	

Supplementary Table 2 shows available disease and patient characteristics for patients diagnosed in May (diagnosis month with subsequent lowest mortality) and November (diagnosis month with subsequent highest mortality). Age was compared using a two-sided T-test. Discrete variables (sex, year of diagnosis, histological subtype, Ann-Arbor stage) were compared using the Chi-square test. NS: Nodular sclerosis. MC: Mixed cellularity. LR: Lymphocyte-rich. LD: Lymphocyte-depleted. NLPHL: Nodular lymphocyte-predominant Hodgkin lymphoma. NOS: Not otherwise specified.
